# Novel Fluoroquinolones with Possible Antibacterial Activity in Gram-Negative Resistant Pathogens: In Silico Drug Discovery

**DOI:** 10.3390/molecules28196929

**Published:** 2023-10-04

**Authors:** Manuel Alejandro Coba-Males, Martin J. Lavecchia, Christian David Alcívar-León, Javier Santamaría-Aguirre

**Affiliations:** 1 Grupo de Investigación en Biodiversidad, Zoonosis y Salud Pública (GIBCIZ), Instituto de Salud Pública y Zoonosis (CIZ), Facultad de Ciencias Químicas (FCQ), Universidad Central del Ecuador, Quito 170521, Ecuador; 2 CEQUINOR (UNLP-CONICET, CCT-La Plata, Associated with CICBA), Universidad Nacional de La Plata, La Plata 1900, Argentina; lavecchia@quimica.unlp.edu.ar; 3 Facultad de Ciencias Químicas (FCQ), Universidad Central del Ecuador, Quito 170521, Ecuador; cdalcivar@uce.edu.ec

**Keywords:** molecular docking, fluoroquinolones, DNA gyrase, bacterial resistance, molecular dynamics simulations, in silico drug discovery

## Abstract

Antibiotic resistance is a global threat to public health, and the search for new antibacterial therapies is a current research priority. The aim of this in silico study was to test nine new fluoroquinolones previously designed with potential leishmanicidal activity against *Campylobacter jejuni*, *Escherichia coli*, *Neisseria gonorrhoeae*, *Pseudomonas aeruginosa*, and *Salmonella typhi*, all of which are considered by the World Health Organization to resistant pathogens of global concern, through molecular docking and molecular dynamics (MD) simulations using wild-type (WT) and mutant-type (MT) DNA gyrases as biological targets. Our results showed that compound **9FQ** had the best binding energy with the active site of *E. coli* in both molecular docking and molecular dynamics simulations. Compound **9FQ** interacted with residues of quinolone resistance-determining region (QRDR) in GyrA and GyrB chains, which are important to enzyme activity and through which it could block DNA replication. In addition to compound **9FQ**, compound **1FQ** also showed a good affinity for DNA gyrase. Thus, these newly designed molecules could have antibacterial activity against Gram-negative microorganisms. These findings represent a promising starting point for further investigation through in vitro assays, which can validate the hypothesis and potentially facilitate the development of novel antibiotic drugs.

## 1. Introduction

In the last decade, bacterial microorganisms have developed resistance mechanisms that have severely limited the effectiveness of conventional antibiotic therapies in treating infectious diseases [1]. The problem of antibiotic resistance has been acknowledged by a United Kingdom committee that reviews resistance data, which has warned that unless the irrational use of antibiotics is controlled, by 2050, it could lead to the death of 10 million people each year worldwide [2].

Data collected by the World Health Organization in 2019 showed that 1.27 million deaths were directly related to antibiotic resistance, mainly due to six pathogens, considered a priority [3]. The increased levels of cross-resistance of antibiotics have led the available drugs to treat bacterial infections begin to present therapeutic limitations [4]; consequently, some conventional antibiotic therapies against infectious diseases are losing effectiveness [4]. Gram-negative bacteria have been reported to develop resistance to various broad-spectrum antibiotics, such as β-lactams, cephalosporins, carbapenems, and fluoroquinolones [5], which are considered clinically significant. In the last decade, resistant Enterobacteriaceae, including *Escherichia coli*, *Pseudomonas*, and *Acinetobacter*, have demonstrated high mortality rates in hospitals due to their multi-drug resistance [3,5].

Given the ongoing challenge of drug-resistant bacterial infections and the associated mortality rates, there is a pressing need to improve the research and development of new antibacterial drugs focused on drug-resistant pathogens [6].

Advances in the digital era have enabled the use of computational approaches to discover and develop new drugs [7]. For example, Ali et al. [7] examined ten molecules derived from a marine fungus through docking studies against mutant transpeptidase in *N. gonorrhoeae*. Alhadrami et al. [8] also conducted in silico studies with anthraquinones from medicinal plant extracts against ligases of *E. coli* multi-drug resistance. Both studies were important in identifying compounds with potential activity against their biological targets. The evidence suggests that molecular docking has become an essential method to estimate the best chemical interactions between a drug and its molecular target [9], with the aim of rapidly identifying new molecules as candidates with therapeutic activity [10]. 

**Figure 1:**
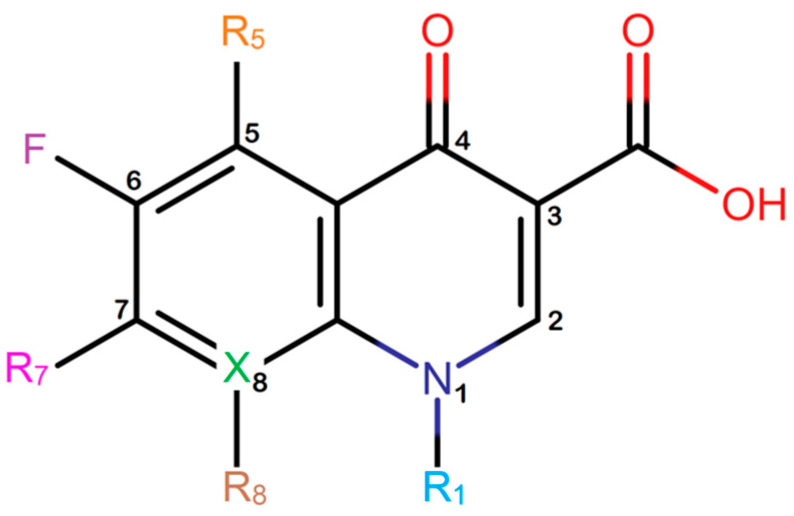
Fluoroquinolone structure. Pharmacophore group (4-quinolone) where the substituents in R_1_, R_5_, R_7_, R_8_, and X (usually corresponds to a C or N atom) could improve the pharmacokinetic or pharmacodynamic of the drug. Images generated with MarvinSketch.

The first fluoroquinolone, norfloxacin, was discovered in 1980 through a structural modification to nalidixic acid, a compound reported by George Y. Lesher in 1962 [11]. Fluoroquinolones (Figure 1) are a group of synthetic antibiotics with a fluorine atom attached at position 6 of the quinolone pharmacophore group [12,13]. Due to their rapid absorption, large volume of distribution, high bioavailability, and long plasma half-life, they are commonly used as first-line therapeutic options for treating various infectious processes [14].

The mechanism of action of fluoroquinolones is based on the specific inhibition of DNA gyrase (for Gram-negative bacteria) or topoisomerase IV (for Gram-positive bacteria) during replication, transcription, and repair of bacterial DNA, which prevents the supercoiling of nucleic acids [15]. DNA gyrase has an active heterotetrameric complex structure A_2_B_2_ [16] composed of two A subunits (GyrA) that are important for substrate recognition, cell targeting, and protein interactions, and two B subunits (GyrB) responsible for providing support for DNA binding [17].

For Gram-negative bacteria, fluoroquinolones bind to DNA gyrase through two subunits of protein in their *N*-terminal, close to Tyr122, which is the protein’s active site. In this area, there is also a quinolone resistance-determining region (QRDR), which allows interactions with the drug to generate a ternary complex. This complex can reversibly inhibit the synthesis of both DNA and mRNA [18]. Chromosomal mutations affect the QRDR of DNA gyrase, and constitute the mechanism most frequently involved in resistance to quinolones among Gram-negative bacteria [19].

In a previous research, nine fluoroquinolones were designed using a computational approach aimed towards leishmanicidal activity [20]. Our research aims to estimate and predict the affinity of these new compounds as antibacterial against Gram-negative bacteria through in silico methodologies in DNA gyrase wild-type (WT) and mutant-type (MT) for *Escherichia coli*, *Campylobacter jejuni*, *Neisseria gonorrhoeae*, *Pseudomonas aeruginosa*, *Salmonella enterica* serovar *typhi*. To compare the affinity of the newly designed fluoroquinolones with existing compounds, we used four FDA-approved fluoroquinolones as controls and calculated their binding energies scores. Furthermore, we performed molecular dynamics (MD) simulations using the MM-GBSA method to evaluate the stability of the ligand with the best binding energy in the ternary complex with DNA and DNA gyrase, and to identify the regions with the highest contribution to the binding enthalpy. 

Based on our in silico findings, we will select the most promising candidates, with the best binding energy, and conduct further studies, including the design of a synthesis route to obtain the molecules in the laboratory for in vitro assays. These experiments will validate the affinity demonstrated through molecular docking and evaluate the compound’s activity, providing critical insights into its potential as an antibacterial drug against drug-resistant pathogens.

## 2. Results

Molecular docking and molecular dynamics simulations were performed with the new nine compounds focused on DNA gyrase of five Gram-negative bacteria. Four fluoroquinolones with known antibacterial activity were used as controls.

### 2.1. Conformation Analyses of New Fluoroquinolones

**Figure 2:**
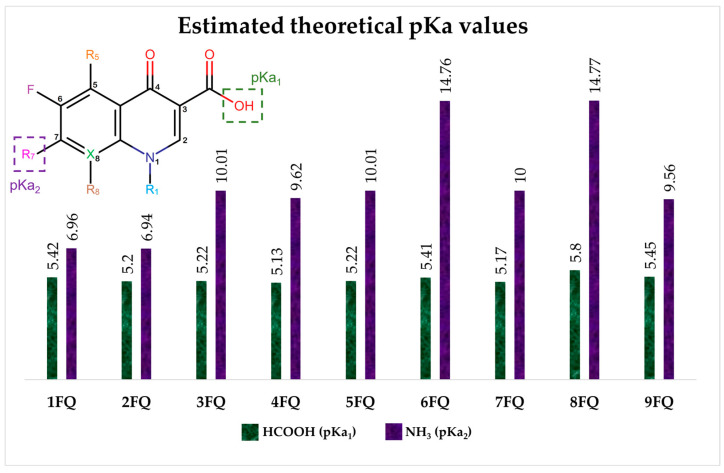
Estimated theoretical pKa values for the new fluoroquinolones (**1FQ** to **9FQ**). pKa_1_ corresponds to the ionization of carboxylic acid to carboxylate at position C_3_, while pKa_2_ is the protonation in the tertiary amine at R_7_ to generate quaternary ammonium salt.

The pharmacophore structure of fluoroquinolones contains ionizable groups with amphoteric behavior sensitive to pH. Theoretical pKa values were estimated using MarvinSketch software for the new molecules, depicted in Figure 2 with a particular focus on the two main functional groups located at the C_3_, which corresponds to the carboxyl group, and on the side chain of the amino group located at R_7_.

The pKa_1_ values for all the compounds were found to be similar, ranging from 5.13 to 5.80. This is because the deprotonation of the carboxylic acid is influenced by the electronegativity of the neighboring atoms at C_2_ and N_1_. On the other hand, the pKa_2_ values range between 6.94 and 14.77 due to the different radicals of tertiary amine over R_7_, which, depending on their structure, can be voluminous groups that generate steric hindrance to attracting one proton.

At neutral pH, we found that the predominant species for **3FQ**, **4FQ**, **7FQ**, and **9FQ** was the zwitterionic form, while the other compounds show predominance of the basic anionic due to carboxylic deprotonation.

From these compounds, the minimum energy structures were obtained through semi-empirical PM6/ZDO theory level using three principal dihedral angles: ϕ_1_ (C_2_-C_3_-C_1′_-R_3′_); ϕ_2_ (C_6_-C_7_-R_7_-R_7′_); and ϕ_3_ (C_2_-N_1_-R_1_-R_1′_), based on potential energy surface plots after a scan of total energy for each dihedral angle. The selected atoms are numbered based on the nomenclature of the pharmacophore group of fluoroquinolones, based on the 4-quinolone structure (4-oxo-1,4-dihydroquinoline).

**Figure 3:**
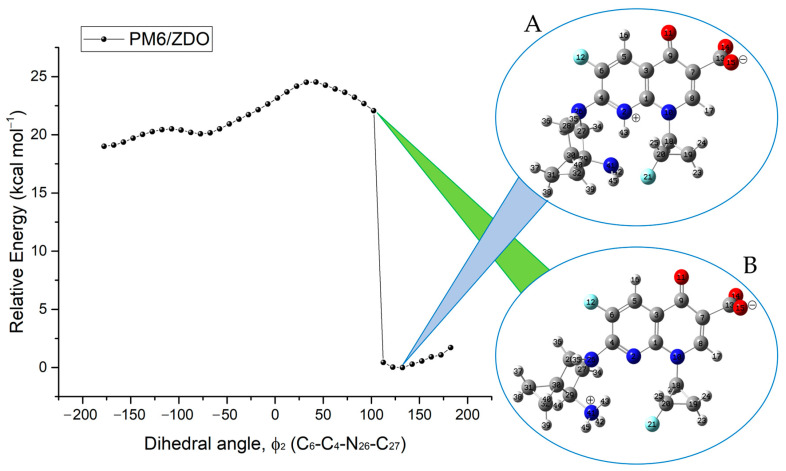
Potential energy curve calculated at the PM6/ZDO level of theory for the internal rotation around the C_4_-N_26_ bond, which corresponds to the dihedral angle ϕ_2_ for **9FQ**. The significant change at approximately 100 degrees reflects the interconversion between tautomers (**A**,**B**).

The graph of relative energy (see Figure 3) after conformational analysis was essential to identify the most stable three-dimensional conformer in each designed molecule.

Potential energy curve for compound **9FQ** shows a jump of about 20 kcal/mol in energy close to 100 degrees in the analyzed dihedral. This transition reveals the presence of two prototropic tautomers. The torsion of the pyrrolidine ring enabled the rearrangement of a proton from the amino substituent of the pyrrolidine to the nitrogen in the fluoroquinolone structure.

### 2.2. Molecular Docking

To estimate the stability of the drug–protein–DNA complex formation, we evaluated the binding of the new compounds to the active site of the targets using modeled structures from the crystal ciprofloxacin (**CPF**)–DNA gyrase–DNA complex at 3.35 Å resolution (PDB ID: 2XCT) for each microorganism. The results presented in Table 1 were based on previous structural information of a crystal system with a known ligand. Additionally, we performed docking assays with four classical fluoroquinolones that are globally commercially available antibiotic drugs for comparative purposes.

Among the newly designed compounds, **9FQ** and **1FQ** exhibited the lowest binding energies scores in all of the tested microorganisms, even compared to **CPF**, **OFX**, **LEV**, and **NOR**.

**Figure 4:**
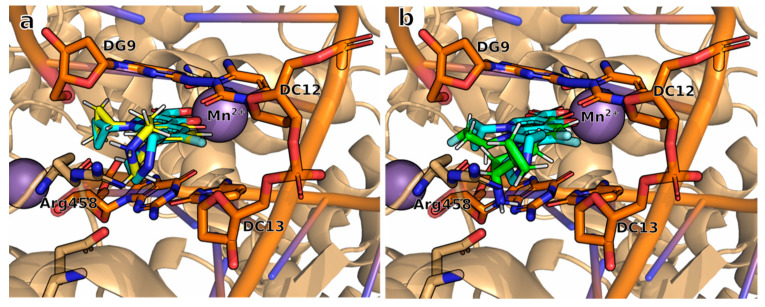
Binding mode of ligand to its receptor in the active site of DNA–DNA gyrase complex for *E. coli* in the assays of (**a**) re-docking using the crystal structure of **CPF** (re-docked structure: **yellow**; crystal structure: **blue**), and (**b**) superposition of **9FQ** docking pose (**green**) with **CPF** crystal structure (**cyan**). Images generated with PyMOL.

To ensure the reproducibility of our docking assays for the new compounds, we conducted a re-docking test using the crystallized **CPF** on the protein with mutations of the residues close to the ligand binding site based on *E. coli*. The ligand **CPF** re-docking in the complex was superimposed on the reference complex as shown in a 3D way, showing similar binding to that experimentally determined in the crystal structure, as shown in Figure 4a. This indicates that the computational approach is able to accurately reproduce the binding interactions.

The positive outcomes obtained from the re-docking experiments provide solid evidence to support the reliability of the docking simulations performed on the new compounds using HYBRID 4.1.1.0. In Figure 4b, we can appreciate that binding for the compound **9FQ** maintains a spatial conformation analogous to the crystallized **CPF**. This pose would enable the interaction with the DNA–DNA gyrase complex.

### 2.3. Molecular Dynamics Simulations

The MD simulations were performed using the complex of *E. coli* with **1FQ** and **9FQ**, which had the best binding energy scores, along with a simulation for **CPF** as a control.

The results of binding energy estimated with the MM-GBSA method are shown in Table 2; these showed a good correlation with the scores obtained from molecular docking using HYBRID.

**Figure 5:**
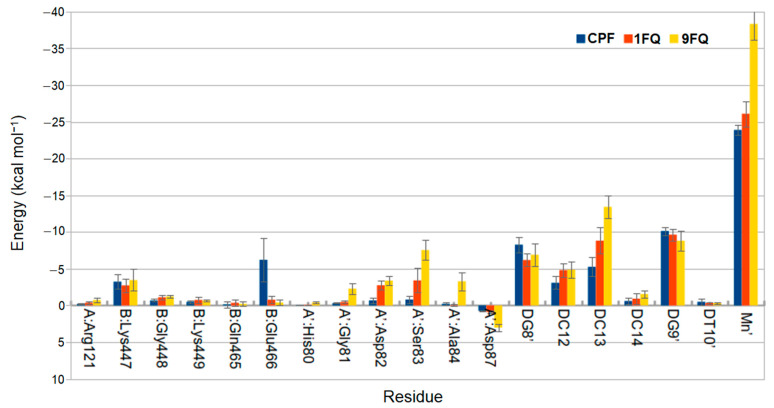
Pair MM–GBSA energy decomposition of **1FQ**, **9FQ**, and **CPF**.

To compare the binding modes between those three ligands, we perform an energy decomposition to identify relevant interactions that occur in the ligand–DNA–receptor complex. The calculations were performed with the MMPBSA.py module with a pairwise energy decomposition scheme. The bar plot in Figure 5 shows those interactions with an energy greater than |0.3 kcal·mol^−1^|; each residue has been renumbered based on GyrA and GyrB sequences for *E. coli* available in UniProt.

**Figure 6:**
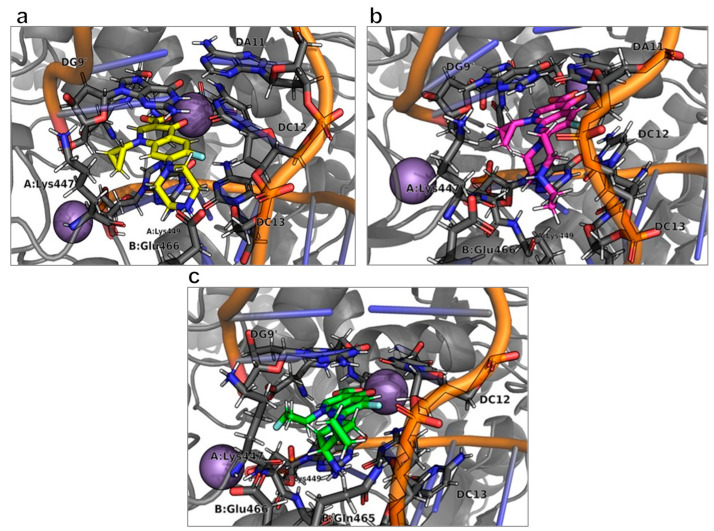
Binding modes of (**a**) **CPF**, (**b**) **1FQ**, and (**c**) **9FQ** correspond to molecular dynamics trajectories. Images generated with PyMOL.

The location of each ligand within the binding site, where interactions with their receptor occur, is depicted in Figure 6. It is noteworthy that the interaction patterns of the three analyzed compounds exhibited remarkable similarity.

**Figure 7:**
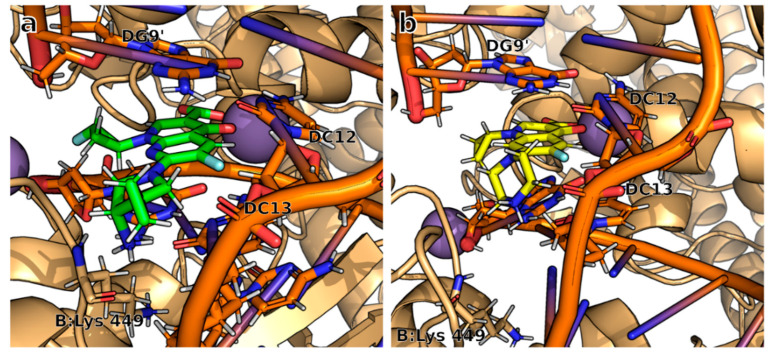
The binding site of (**a**) **9FQ**, and (**b**) **CPF** shows the structural difference in the binding mode with the DNA–DNA gyrase complex. Images generated with PyMOL.

Upon closer examination, we identified notable differences in the interaction patterns between **9FQ** and **CPF** within the GyrB chain, particularly with regard to amino acid Lys449 and nucleotide DC13. This interaction with **9FQ** was more favorable (Figure 7). 

**Figure 8:**
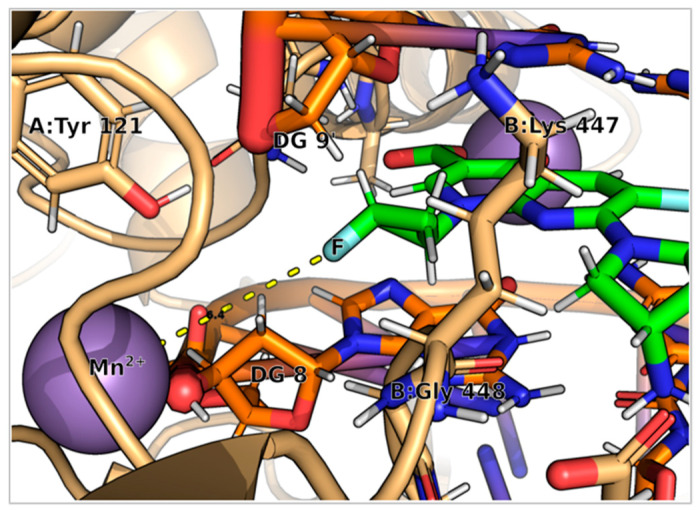
Interaction between the fluorine of the cyclopropyl group in the **9FQ** structure and the Mn^2+^ cation of DNA gyrase enables the binding of the fluoroquinolone to the enzyme for complex stabilization with DNA. Image generated with PyMOL.

However, the presence of the cyclopropyl group in **9FQ** did not result in an improvement in binding energy score, as observed in the interaction generated in the binding. Therefore, future works will consider the possibility of analyzing structural substitutions with larger or elongated groups to improve the binding affinity (Figure 8).

**Figure 9:**
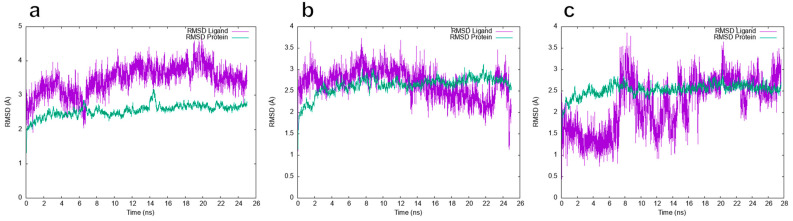
Analysis of RMSD trajectories for the ligands (purple color) (**a**) **9FQ**, (**b**) **1FQ**, and (**c**) **CPF** versus protein complexes (turquoise color) throughout the all-atom molecular dynamic simulation.

Additionally, trajectory analysis for 25 ns allowed us to calculate the root mean square deviation (RMSD) at **9FQ**, **1FQ**, and **CPF** to determine the convergence and stability of the simulations (see Figure 9). The MD simulations at 300 K for **9FQ** appear to exhibit stability from 8 ns without significant changes. In contrast, for **CPF**, higher fluctuations are observed throughout the entire trajectory.

### 2.4. Toxicity Prediction

We obtained in silico predictive results that can be used as a reference for the toxicity of these two molecules (see Table 3). However, toxicity evaluation must be performed through in vitro assays to obtain actual data about the compounds and establish therapeutic doses for further studies.

### 2.5. Lipinski’s Five Rules

The best molecules satisfied Lipinski’s rule of 5, which is one of the essential criteria to predict oral drug likeliness. The results for Lipinski’s rule of 5 were obtained from **1FQ** and **9FQ**, as they were the compounds that showed the best affinity with DNA gyrase (see Table 4).

Additionally, the synthetic accessibility for compounds **1FQ**, and **9FQ** was 3.68 and 3.90, respectively, and these values were estimated in SwissADME server.

## 3. Discussion

In this study, we evaluated the affinity of nine new fluoroquinolones against DNA gyrase in resistant Gram-negative pathogens with clinical relevance using in silico techniques such as molecular docking and molecular dynamics simulations. 

Antibiotic resistance is still a significant world problem which keeps leading to daily clinical cases of infections caused by resistant bacteria [21]. The World Health Organization has included some antibiotics in its essential medicines list, which has first- or second-choice antibiotics that are the best therapeutic option; however, some of them were catalogued as more prone to be a target of antibiotic resistance [22]. In this way, healthy authorities try to incentive the research and development of new drugs with antimicrobial activity that help in the treatment of infectious diseases, with a particular focus on pathogens considered to be priorities by the World Health Organization [23]. 

Computational models have become an important tool to carry out in silico studies to find out the most stable, specific, and favorable pose between a ligand and its biological target [24]. The drug design through in silico models is a new paradigm with a positive impact on the process of drug discovery [25]. Thus, with these models, it is possible to establish early studies to try to understand if there are possibilities that a new molecule would have any pharmacological effect due to interaction with a receptor that involves biological changes [26]. Nevertheless, this study must only be considered an initial approximation that requires a second stage that will perform in vitro assays to confirm or discard in silico results.

Fluoroquinolones are considered ideal antibiotics due to their favorable properties’ pharmacodynamics, pharmacokinetics, and minimal adverse effects. Although, their passive diffusion via porins or diffusion through cell membranes implies complex processes that involve physical and chemical factors [15,27]. The efforts to synthesize novel compounds that are derivatives of fluoroquinolones to evaluate their antibacterial activity have grown in the last decade. All of this is a consequence of the levels of concern regarding resistance to this antibiotic group, especially in bacteria such as *E. coli*, *K. pneumoniae*, *P. aeruginosa*, and *A. baumannii* [28,29,30,31,32,33].

Our in silico study focused on evaluating the potential of nine compounds, previously designed using a de novo approach described in an undergraduate thesis, to inhibit DNA gyrase in Gram-negative pathogens [20]. We began by analyzing the ionized molecules at pH 7.0. Fluoroquinolones are amphoteric in nature, and four of the compounds (**3FQ**, **4FQ**, **7FQ**, and **9FQ**) showed a zwitterionic form. Despite the fact that zwitterions have a significant dipole at physiological pH [34], the non-ionized forms are generally more suitable for diffusion through the lipid bilayer of cell membranes [27]. Nonetheless, certain zwitterionic antibiotics such as tetracyclines and fluoroquinolones [35], have demonstrated moderate-to-high passive permeability in vitro and favorable bioavailability in vivo [36,37].

Docking results indicate the lowest binding energy for **9FQ**, with a closer average value of −14.0 kcal/mol in wild and mutant-type bacteria. That suggests a good affinity of **9FQ** to the active site of protein DNA gyrase if we compare with binding energy values obtained with ciprofloxacin, norfloxacin, levofloxacin, and ofloxacin, which were in the range between −9.0 and −12.0 kcal/mol. The values showed for these standard fluoroquinolones are similar to those of other studies, where the binding energy of ciprofloxacin against DNA gyrase of *S. aureus* was −12.8 kcal/mol [38], and the low binding energy obtain against DNA gyrase of *E. coli* barely reached a value of −6.4840 kcal/mol [39]. Additionally, compound **1FQ** also showed an average binding energy close to −13.0 kcal/mol. The binary complex (DNA–DNA gyrase) seems to have a good affinity with any of the two new compounds; however, the question as to which of these bindings is more stable than the other remains. The answer to that question required a brief analysis through molecular dynamics simulations with the **1FQ**, **9FQ,** and **CPF** compounds. The MD simulations for 25 ns allowed us to estimate the binding free energy as the difference between the bound and unbound states of the protein and the ligand [40]; the values were −40.1 ± 4.0, −45.2 ± 4.8, and −35.8 ± 3.7 kcal/mol for **1FQ**, **9FQ**, and **CPF**, respectively, with DNA gyrase of *E. coli* because it was the protein of the organism with the best score in the docking assays. 

MD simulations provide clear information about the structural changes of a system throughout time, and have been used as a method for the study of drugs [41,42]. Our interest was precisely focused on understanding the binding mode between our ligand and the target to know the principal molecular interactions that enable the stabilization of the DNA–DNA gyrase complex. The most relevant molecular interaction occurs through the chelation of a noncatalytic manganese ion against the DNA–DNA gyrase complex; this enables a relevant interaction between the compounds with enzyme residues and the DNA chain. During the binding of compound **9FQ** with the DNA and DNA gyrase, we found and highlight that His80, Gly81, Asp82, Ser83, Asp84, and Asp87 were the residues in the GyrA chain that interacts with functional groups of this molecule. All of these amino acids are in the QRDR, a region that has been proposed as a potential binding site to the fluoroquinolones [43]; mutations at this site have also been associated with increases in resistance levels. Another important interaction was observed in Arg121, which, due to its proximity to the active site of the protein, could play an important role in reversibly canceling the replication of DNA caused by an exogenous ligand [18]. On the other hand, interactions with Lys447, Gly448, Lys449, Gln465, and Glu466 residues of the GyrB chain would also be implicated in blocking DNA replication. Although these sites are distal to the active site, the binding with the amino acids inside TOPRIM domain of GyrB generates conformational changes in DNA gyrase that inhibit its activity [44]. The interaction with Lys449 of the GyrB chain in the **9FQ** compound was more favorable to take place through the amino group of the tetrahydropyrrole, which is closer compared to the piperazine group of **CPF**. This structural difference, together with the presence of fluorine in the cyclopropyl ring in **9FQ,** would enable a better interaction in the complex. This is reflected in the convergence and stability shown by the ligand in the RMSD plot, where at least the first 25 ns of the simulation seem to be stable. Thus, for this reason, we do not consider these results an absolute truth, but an approximation to continue in vitro experimental studies to determine the antibacterial activity of this new compound that we propose here.

The molecules have not yet been synthesized, and the in silico results that we have obtained suggest a good affinity against DNA gyrase. These compounds also comply with the established parameters in Lipinski’s rule; therefore, there is a good relationship between the solubility and the ability of the molecules that could diffuse through biological membranes in a hypothetical absorption process. Additionally, we assigned a complexity score in the synthesis of the compounds; the results were 3.68 and 3.90 out of a maximum of 10, for **1FQ** and **9FQ,** respectively; this means that, initially, we would not expect to have high complexity to start designing a synthesis route that allows us to obtain the molecules at laboratory level for in vitro evaluations.

## 4. Materials and Methods

**Figure 10:**
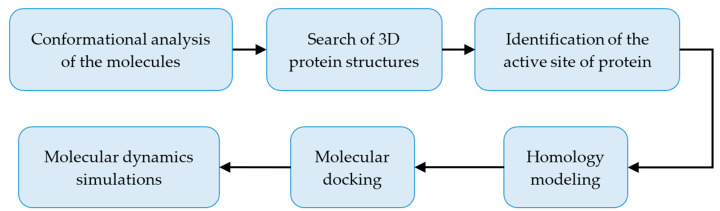
General workflow performed in the present study.

Briefly, Figure 10 summarizes the workflow carried out.

### 4.1. Preparation of Ligands

Ligand structures were built with Avogadro 1.2.0 [45]. First, using MarvinSketch 21.12.0. software [46], we generated the microspecies distribution curve as a function of pH for the nine compounds; then, the software estimated their theoretical pKa. The structure of the predominant species at pH 7.0 for each molecule was subjected to an energy optimization using the MMFF94 force field in Avogadro 1.2.0 [45]. After that, we used Gaussian 09W [47] to carry out a conformational analysis based on a semi-empirical method with PM6/ZDO theory level. All calculations were carried out in the absence of a solvent.

Table 5 shows the nomenclature and two-dimensional structures of the new compounds that have been used in the present study.

Additionally, we used ciprofloxacin (**CPF**), ofloxacin (**OFX**), levofloxacin (**LVF**), and norfloxacin (**NOR**) as controls. These fluoroquinolones were prepared by following the same process.

### 4.2. Retrieval of DNA Gyrase Structures

The structures of DNA gyrase for the five Gram-negative pathogens were modeled using the crystal structure of *Staphylococcus aureus* gyrase complex with ciprofloxacin and DNA (accession ID: 2XCT) [48] as a template, obtained from the RCSB Protein Data Bank in PDB format (https://www.rcsb.org/) (accessed on 1 September 2022) [49]. For the wild-type structures, the sequences were retrieved from UniProt (https://www.uniprot.org/) (accessed on 8 September 2022) [50], see Table 6.

To construct the 3D structure of DNA gyrase of mutant type for each bacterium, we considered mutations associated with the resistance to fluoroquinolones. These changes focus on the GyrA subunit over the quinolone resistance-determinate region (QRDR), see Table 7.

### 4.3. Preparation of Molecular Systems

**Figure 11:**
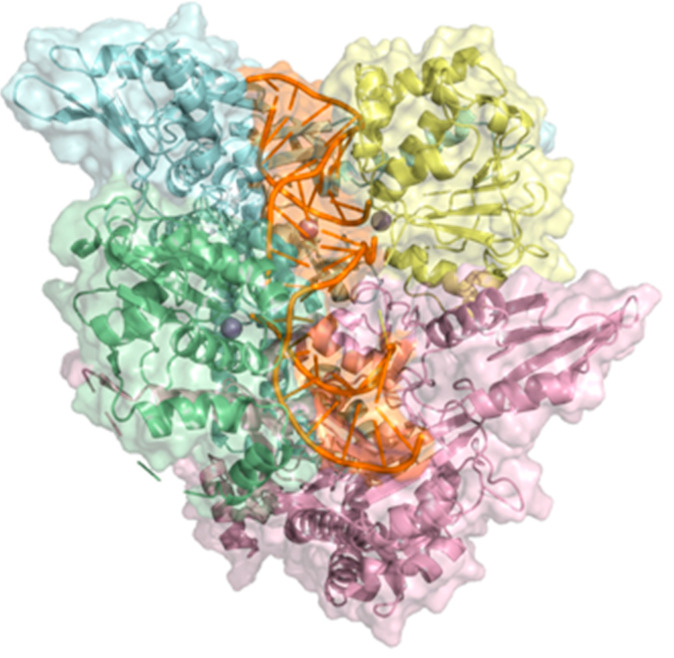
Part of the 2XCT crystal structure is considered for molecular docking and molecular dynamics simulations. GyrB (green), GyrA (light blue), GyrB′ (yellow), GyrA′ (pink), DNA fragment (orange), Mn^2+^ (gray spheres). Figure created with PyMOL.

The simulations were based on a section of the X-ray crystal structure of PDB ID: 2XCT [48] available in Protein Data Bank [49] (see Figure 11). The portion of this structure was considered for molecular docking and molecular dynamics simulations. The preparation of systems was carried out with Chimera [56]. Manganese ions were conserved, while the water molecules were removed. It should be clarified that the crystallized ion in the PDB 2XCT structure was retained; however, in the WT system, this cation corresponds to magnesium. Hydrogen atoms were added following the hydrogen bonding pattern. Nearby residues (up to 5 Å) of the **CPF** in their original position were replaced with Chimera to reproduce the wild-type and mutated sequences of the pathogens studied.

### 4.4. Molecular Docking

Molecular docking studies were performed to find and score the complex between DNA gyrase–DNA–ligand binding poses using HYBRYD 4.1.1.0 [57], with the **CPF** of chain H in the crystal structure as a reference ligand. This feature of pre-aligning the ligands to a reference ligand of the crystal structure allows dockings to be performed in a complex system that includes, in addition to protein, DNA and cations. OMEGA 4.1.2.0 [58] was used to explore and generate 3D ligand conformers of each compound with the “pose” option, while other parameters were set to their default values. A re-docking assay was carried out to reproduce the ligand binding in the crystallized structure under the same parameters and pocket that was considered for new compounds. Finally, the best conformations were analyzed using PyMOL [59].

### 4.5. Molecular Dynamics Simulations

Molecular dynamics (MD) simulations were performed with NAMD 2.13 [60]. We prepare input files by protonating, renumbering atoms, and separating the complex, ligand, and receptor structures. Then, the topology files were assembled, and the solvation and energy minimization of the entire system was performed. Next, the temperature and pressure were equilibrated to finally run the molecular dynamics simulations.

The protein and DNA were described using the Amber14SB [61] and OL15 [62] force fields, respectively. The ligands were described using the generalized amber force field [63] with charges derived from AM1BCC, which were calculated with the antechamber module.

Leap and antechamber are included in the package AmberTools 22 [64]. The whole system (gyrase complex, DNA, Mn^2+^ cations, and ligand) has a negative net charge; therefore, sodium cations were added as counterions with the leap module to achieve electroneutrality. The neutralized systems were immersed in a box of TIP3P waters that extended up to 15 Å from the solute.

Contributions 1–4 were multiplied by the factor 0.83 to meet AMBER force field requirements. Van der Waals interaction cut-off distances were set to 12 Å and long-range electrostatic forces were calculated with the Ewald summation method of particle mesh at 1.0 Å grid size. The system received 10⁵ steps of minimization, heating from 0 to 310 K in 30 ps, and 25 ns of equilibrium/production simulation.

The trajectory length was chosen based on the relatively large size of the entire system, including the explicit solvent (more than 200,000 atoms). In all equilibrium/production simulations, the temperature was kept constant (310 K) using Langevin dynamics with a damping coefficient of 5 ps^−1^, while the pressure was kept constant at 1 atm through the piston method. Nosé-Hoover Langevin had a decay period of 200 fs and a decay time constant of 100 fs. The hydrogen atom bonds of the waters were constrained with the SHAKE algorithm. A 1 fs time step was also performed throughout molecular mechanics. RMSD values were plotted to estimate the convergence and stability of the simulations.

The free binding energies of the ligands with the DNA gyrase–DNA complex were calculated using the MM-GBSA method.

The free energy of solvation was calculated with the Born (GB) model using igb = 5 as the selected model of the MMPBSA.py module [65] The hydrophobic contribution was calculated with the surface area accessible to the solvent. Ligand binding free energies were accumulated with a single track over 100 snapshots taken from the last 10 ns portion of the molecular dynamic simulation tracks.

The representation of the interactions was carried out under the free energy decomposition analysis for the total binding free energies in the pairs of ligand–amino acid or –nucleotide, and the calculations were performed with a pairwise energy decomposition scheme (ide-comp option 3) of the MMPBSA.py module.

### 4.6. Toxicity in Silico Prediction

Toxicity prediction was carried out with the ProTox-II webserver (https://tox-new.charite.de/protox_II/) (accessed on 14 January 2023) [66]. We predicted the oral toxicity class in the compounds with the best binding energy in the formation of the ternary complex.

### 4.7. Lipinski’s Five Rule Estimation

We used the SwissADME web tool (http://www.swissadme.ch/) (accessed on 16 January 2023) [67] to predict the druglike nature of the new fluoroquinolones that showed the best binding affinity to the biological target. This estimation is a way of supporting drug discovery to recognize whether the ligand could have the optimal chemical and physical properties to be orally bioavailable.

## 5. Conclusions

Since antibiotic resistance is still a public health concern that has been aggravated by, among other causes, the lack of development of effective drugs with antibacterial activity in recent years, discovering new therapeutic agents is a necessity. The present study used computational methodologies to investigate and evaluate the affinity of nine new fluoroquinolones to binding the DNA gyrase in Gram-negative pathogens and inhibit its activity. Compound **9FQ** seems to be a promising compound with antibacterial activity against DNA gyrase due to its good affinity and stability in the formation of the complex with DNA and DNA gyrase. Further in vitro and in vivo experiments must be performed to guarantee the desired activity without representing a health risk.

## Figures and Tables

**Table 1:** Predicted binding energy score (kcal·mol^−1^) during drug–gyrase–DNA complex formation.

Ligand	DNA Gyrase							
	Binding Energy Score (kcal/mol)							
	*E. coli*		*P. aeruginosa*		*C. jejuni*		*S. typhi*	*N. gonorrhoeae*
	WT	MT	WT	MT	WT	MT	MT	MT
**1FQ**	−13.6	−14.0	−13.5	−13.5	−14.4	−13.6	−14.2	−13.7
**2FQ**	−8.7	−9.0	−8.7	−8.8	−8.0	−7.4	−9.9	−8.7
**3FQ**	−11.6	−10.1	−12.4	−10.3	−10.7	−10.4	−11.9	−11.6
**4FQ**	−11.6	−12.2	−11.6	−11.6	−11.2	−9.7	−12.9	−11.7
**5FQ**	−11.8	−12.0	−11.5	−11.4	−12.0	−11.7	−12.6	−11.8
**6FQ**	−10.9	−11.0	−10.6	−10.6	−11.1	−10.6	−11.5	−11.2
**7FQ**	−10.7	−10.8	−10.4	−10.5	−11.1	−10.6	−11.4	−10.8
**8FQ**	−12.0	−11.6	−11.2	−11.1	−12.0	−11.2	−12.0	−12.2
**9FQ**	−14.4	−13.6	−13.6	−13.5	−14.2	−13.0	−14.4	−14.3
**CPF**	−12.0	−12.0	−11.5	−11.5	−12.1	−10.9	−12.4	−12.0
**OFX**	−9.6	−9.2	−9.4	−9.4	−10.3	−9.0	−10.0	−9.8
**LEV**	−10.5	−10.0	−9.6	−9.6	−10.0	−9.0	−10.3	−10.6
**NOR**	−11.7	−11.7	−11.3	−11.3	−11.9	−11.5	−12.0	−11.9

WT: Wild-type; MT: Mutant-type; CPF: Ciprofloxacin, OFX: Ofloxacin, LEV: Levofloxacin, NOR: Norfloxacin.

**Table 2:** Total MM–GBSA binding energy with *E. coli* DNA gyrase complex.

Ligand	Binding Energy (kcal·mol^−1^)
**1FQ**	−40.1 ± 4.0
**9FQ**	−45.2 ± 4.8
**CPF**	−35.8 ± 3.7

**Table 3:** Oral toxicity prediction for compounds **1FQ**, **9FQ**, and **CPF**.

Ligand	Predicted LD50 (mg/kg)	Predicted Toxicity Class	Prediction Accuracy (%)
**1FQ**	1866	4	72.9
**9FQ**	2000	4	72.9
**CPF**	2000	4	100

**Table 4:** Lipinski properties for compounds **1FQ**, **9FQ**, and **CPF**.

Ligand	Molecular Weight in g/mol (<500 Da)	Log P (<5)	H-Bond Donor (<5)	H-Bond Acceptor (<10)	Molar Refractivity (<130)
**1FQ**	378.37	2.51	0	7	100.86
**9FQ**	378.37	2.34	2	7	98.44
**CPF**	331.34	2.24	2	5	95.25

**Table 5:** Structure and nomenclature for the newly designed molecules.

Ligand	IUPAC Name	Structure
**1FQ**	7-(4-ethylpiperazin-1-yl)-6-fluoro-1-[(1R,2S)-2-fluorocyclopropyl]-4-oxo-1,4-dihydro-1,8-naphthyridine-3-carboxylic acid	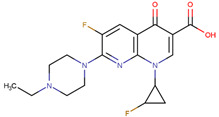
**2FQ**	1-(2,4-difluorophenyl)-7-(4-ethylpiperazin-1-yl)-6-fluoro-4-oxo-1,4-dihydro-1,8-naphthyridine-3-carboxylic acid	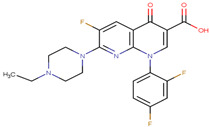
**3FQ**	7-(4-aminopiperidin-1-yl)-1-(2,4-difluorophenyl)-6-fluoro-4-oxo-1,4-dihydro-1,8-naphthyridine-3-carboxylic acid	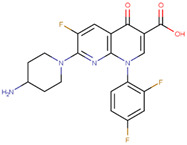
**4FQ**	1-(6-amino-3,5-difluoropyridin-2-yl)-7-[(1R,5S,6R)-6-amino-3-azabicyclo [3.1.0]hexan-3-yl]-6-fluoro-4-oxo-1,4-dihydro-1,8-naphthyridine-3-carboxylic acid	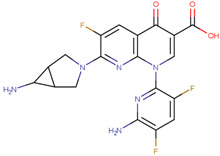
**5FQ**	1-(2,4-difluorophenyl)-6-fluoro-7-(3-hydroxyazetidin-1-yl)-4-oxo-1,4-dihydro-1,8-naphthyridine-3-carboxylic acid	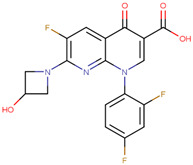
**6FQ**	1-(6-amino-3,5-difluoropyridin-2-yl)-6-fluoro-7-(3-hydroxyazetidin-1-yl)-4-oxo-1,4-dihydro-1,8-naphthyridine-3-carboxylic acid	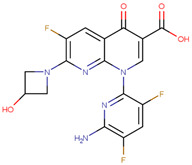
**7FQ**	1-(6-amino-3,5-difluoropyridin-2-yl)-7-[(5S,7S)-7-amino spiro [2.4]heptan-5-yl]-8-chloro-6-fluoro-4-oxo-1,4-dihydroquinoline-3-carboxylic acid	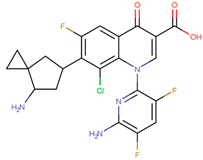
**8FQ**	8-chloro-6-fluoro-1-[(1S,2S)-2-fluorocyclopropyl]-7-(3-hydroxyazetidin-1-yl)-4-oxo-1,4-dihydroquinoline-3-carboxylic acid	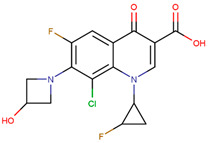
**9FQ**	7-[(7R)-7-amino-5-azaspiro[2.4]heptan-5-yl]-6-fluoro-1-[(1S,2S)-2-fluorocyclopropyl]-4-oxo-1,4-dihydro-1,8-naphthyridine-3-carboxylic acid	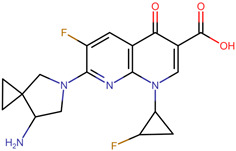

**Table 6:** The accession number of amino acid sequences for the GyrA and GyrB subunits.

Microorganism	GyrA	GyrB
*Staphylococcus aureus*	Q99XG5	P66937
*Campylobacter jejuni*	Q03470	O87667
*Escherichia coli*	P0AES4	P0AES6
*Neisseria gonorrhoeae*	P48371	P22118
*Pseudomonas aeruginosa*	P48372	Q9I7C2
*Salmonella typhi*	P37411	P0A2I4

Sequences for *S. aureus* were used as a template for the modeling of DNA gyrases in other microorganisms.

**Table 7:** Mutations in the GyrA subunit of DNA gyrase.

Microorganism	Mutation	Reference
*Campylobacter jejuni*	Thr86Ile Asp90Asn	[51]
*Escherichia coli*	Ser83Leu Asp87Asn Ala93Gly	[52]
*Neisseria gonorrhoeae*	Ser91Phe Asp95Gly	[53]
*Pseudomonas aeruginosa*	Asp87Asn Thr83Ile	[54]
*Salmonella typhi*	Ser83Phe Asp87Asn	[55]

## Data Availability

All data generated or analyzed during this study are included in this published article.
